# Enzymatic Relay Mechanism Stimulates Cyclic GMP Synthesis in Rod Photoresponse: Biochemical and Physiological Study in Guanylyl Cyclase Activating Protein 1 Knockout Mice

**DOI:** 10.1371/journal.pone.0047637

**Published:** 2012-10-17

**Authors:** Clint L. Makino, Xiao-Hong Wen, Elena V. Olshevskaya, Igor V. Peshenko, Andrey B. Savchenko, Alexander M. Dizhoor

**Affiliations:** 1 Department of Ophthalmology, Massachusetts Eye and Ear Infirmary and Harvard Medical School, Boston, Massachusetts, United States of America; 2 Department of Basic Sciences and Pennsylvania College of Optometry, Salus University, Elkins Park, Pennsylvania, United States of America; University Zürich, Switzerland

## Abstract

Regulation of cGMP synthesis by retinal membrane guanylyl cyclase isozymes (RetGC1 and RetGC2) in rod and cone photoreceptors by calcium-sensitive guanylyl cyclase activating proteins (GCAP1 and GCAP2) is one of the key molecular mechanisms affecting the response to light and is involved in congenital retinal diseases. The objective of this study was to identify the physiological sequence of events underlying RetGC activation *in vivo*, by studying the electrophysiological and biochemical properties of mouse rods in a new genetic model lacking GCAP1. The GCAP1^−/−^ retinas expressed normal levels of RetGC isozymes and other phototransduction proteins, with the exception of GCAP2, whose expression was elevated in a compensatory fashion. RetGC activity in GCAP1^−/−^ retinas became more sensitive to Ca^2+^ and slightly increased. The bright flash response in electroretinogram (ERG) recordings recovered quickly in GCAP1^−/−^, as well as in RetGC1^−/−^GCAP1^−/−^, and RetGC2^−/−^GCAP1^−/−^ hybrid rods, indicating that GCAP2 activates both RetGC isozymes *in vivo*. Individual GCAP1^−/−^ rod responses varied in size and shape, likely reflecting variable endogenous GCAP2 levels between different cells, but single-photon response (**SPR**) amplitude and time-to-peak were typically increased, while recovery kinetics remained faster than in wild type. Recovery from bright flashes in GCAP1^−/−^ was prominently biphasic, because rare, aberrant SPRs producing the slower tail component were magnified. These data provide strong physiological evidence that rod photoresponse recovery is shaped by the sequential recruitment of RetGC isozyme activation by GCAPs according to the different GCAP sensitivities for Ca^2+^ and specificities toward RetGC isozymes. GCAP1 is the ‘first-response’ sensor protein that stimulates RetGC1 early in the response and thus limits the SPR amplitude, followed by activation of GCAP2 that adds stimulation of both RetGC1 and RetGC2 to speed-up photoreceptor recovery.

## Introduction

Guanylyl cyclase activating proteins (GCAP) play an essential physiological role in photoreceptors by accelerating the recovery of rods and cones from excitation by light. Photon absorption by rhodopsin triggers hydrolysis of cGMP and closes cGMP-gated cation channels in the rod plasma membrane, resulting in membrane hyperpolarization (reviewed in [1]–[2]). During the recovery phase of the response to a photon, cGMP levels are restored by retinal membrane guanylyl cyclase (**RetGC**), under the control of Ca^2+^ sensing, guanylyl cyclase activating proteins (**GCAP**s) [3]–[5]. In darkness, high intracellular Ca^2+^ levels promote the binding of Ca^2+^ to GCAPs, which then inhibit cGMP production, but when intracellular free Ca^2+^ is lowered by illumination, Mg^2+^ replaces the Ca^2+^ bound to GCAPs [6]. With Mg^2+^ bound, GCAPs stimulate RetGC to synthesize cGMP at a faster rate. Rods of all vertebrate species express two guanylyl cyclases, RetGC1 and RetGC2 [7]–[8]), as well as two homologous GCAPs – GCAP1 and GCAP2 [4]–[5], [9]–[10]. Additional GCAP isoforms are expressed in the retinas of many species [11]–[13], but GCAP1 and GCAP2 are found in the rods of all vertebrate classes. GCAPs are essential for timely photoresponse recovery and light adaptation, because deletion of the tail-to-tail oriented pair of genes coding for GCAPs 1 and 2 increases the amplitude and prolongs the duration of flash responses in mouse rods and cones [14]–[16]. The two ubiquitous GCAP isoforms have different sensitivities to Ca^2+^ – lower in GCAP1 and higher in GCAP2 [13], [17]–[19]. It has therefore been hypothesized [14], [18], [20] that GCAPs shape the rod photoresponse by activating RetGC in a stepwise, or “relay” [21] fashion: GCAP1 acts first and then GCAP2. Arguing in favor of this model, elimination of GCAP2 produces overall decrease in Ca^2+^ sensitivity of RetGC regulation in the retina. The SPR amplitude does not change but the kinetics of the recovery slow in GCAP2^−/−^ rods [20]. However, the relay mechanism of Ca^2+^ feedback to RetGC in rod physiology could not be decisively validated without knowing how a selective disruption of *GUCA1A* gene coding for GCAP1 affects RetGC regulation and rod photoresponses. The presence of two different isozymes of RetGC – RetGC1 and RetGC2– further complicates understanding of the cGMP synthesis regulation in living photoreceptors. Although our recent study [22] argues that GCAP1 *in vivo* preferentially targets the RetGC1 isozyme, the *in vivo* specificity of GCAP2 toward a particular RetGC isozyme remains unclear.

By studying biochemical and physiological changes caused by elimination of GCAP1, we here demonstrate that even in the absence of the less Ca^2+^-sensitive isoform, GCAP1, the remaining GCAP2 is able to maintain RetGC regulation in GCAP1^−/−^ rods, albeit making it more sensitive to inhibition by Ca^2+^ (and consequently less sensitive to activation by depletion of Ca^2+^). The shape of their photon response shows that GCAP1 is essential for activation of the cyclase early in the course of the response and that restraint of the response amplitude and acceleration of the recovery kinetics in the rods is indeed achieved through the sequential regulation of RetGC activity by the two GCAPs. Moreover, the recovery from a bright flash of hybrid GCAP1^−/−^ rods lacking one of the RetGC isozyme reveals that GCAP2 can effectively provide feedback to either RetGC isozyme *in vivo*. These findings allow us, for the first time, to reconstruct the sequence of the events underlying activation of cGMP synthesis with regard to the role of each GCAP isoforms and each RetGC isozyme in shaping the phases of photoresponse recovery. This study explains how complex relationships between sensor proteins and their target enzymes in a multi-component biochemical pathway tune the physiological function of the rod photoreceptor cell.

## Materials and Methods

### Ethics Statement

All animal procedures were approved by IACUC protocols AAMD0204 from Salus University or 95-06-006 from the Massachusetts Eye and Ear Infirmary, in compliance with NIH guidelines. In the experiments described below, mice of both sexes were used indiscriminately.

### GUCA1A Gene Disruption

**Figure 1:**
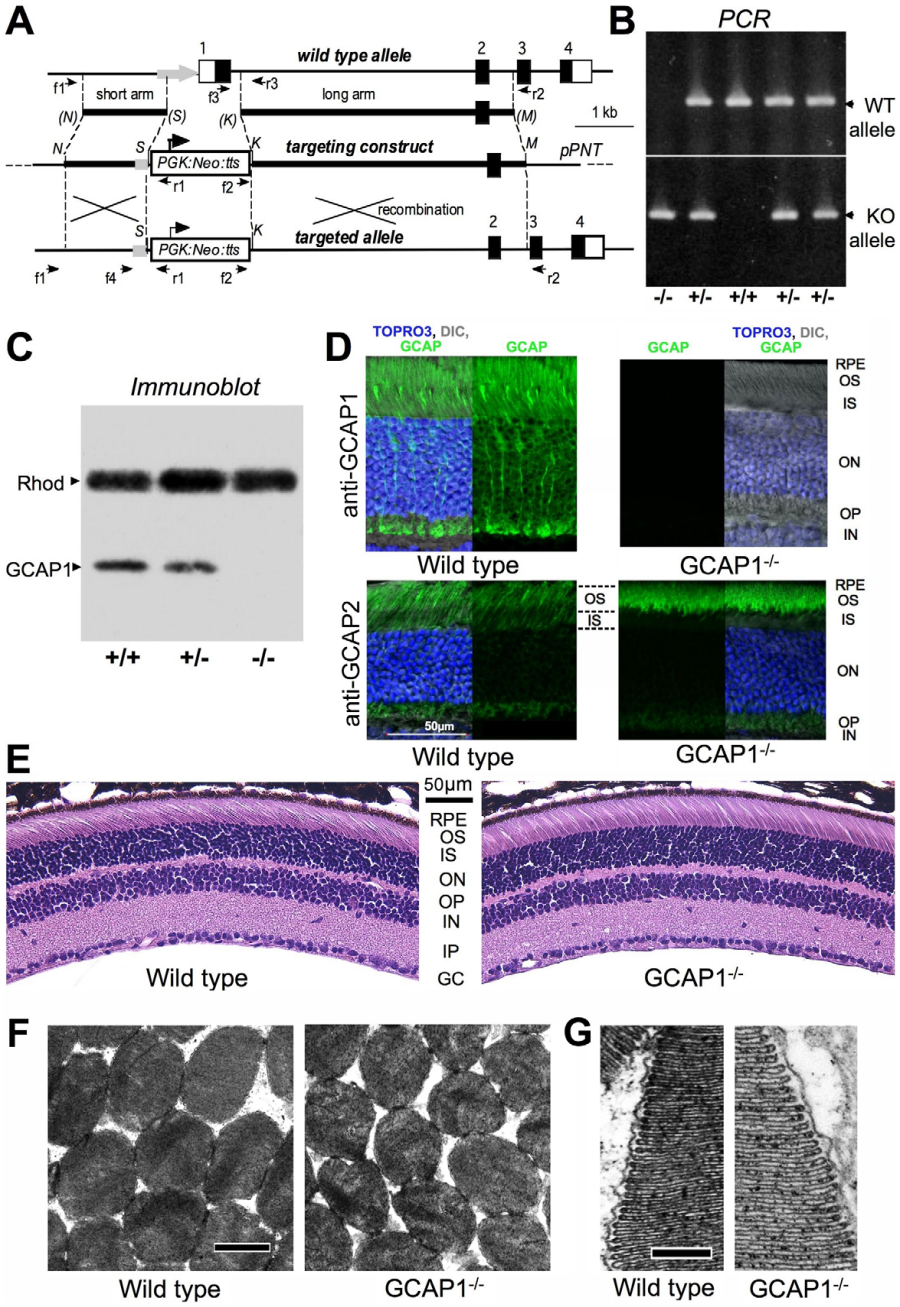
Strategy for GCAP1 gene disruption. **A.** Schematic of the mouse *GUCA1A* gene disruption. The targeting construct was made by inserting the *PGK:Neo:tts* cassette [23] between PCR-amplified 1.5-kb and 5-kb arms to replace the first exon of the *GUCA1A* gene together with the putative promoter region and a part of the first intron as described in detail in Materials and Methods. *K, M, N,* and *S* designate KpnI, MluI, NotI and SbfI restriction sites, respectively; *tts* – transcription termination site in *PGK:Neo* cassette. **B.** PCR products of WT allele (*top*) and the targeted KO allele (*bottom*), amplified from mouse tail DNA from littermates produced by breeding of GCAP1^+/−^ parents using f3 (5′-CCTTGTGCAGGGGACATTAGAAAATAAG) and r3 (5′-CATCTGTTCCACATA CTGGCTGGCT) primers or f2 (5′- TGATATTGCTGAAGAGCTTGGCGGCGAAT) and r3 primers, respectively. **C.** Immunoblotting of SDS polyacrylamide gels containing retina samples from WT, GCAP1^+/−^, and GCAP1^−/−^ littermates simultaneously probed with anti-rhodopsin and anti-GCAP1 polyclonal antibody. Retinas were homogenized in SDS sample buffer on ice and were not boiled prior to electrophoresis, in order to preserve rhodopsin monomer. **D.** GCAP immunofluorescence in retina cryosections from WT (*left panels*) and GCAP1^−/−^ (*right panels*) mice probed with anti-GCAP1 (*upper panels*) or anti-GCAP2 (*bottom panels*) polyclonal antibody and developed with goat-anti rabbit FITC-labeled antibody. WT and GCAP1^−/−^ retinas were fixed, processed and probed with each antibody under identical conditions; images were taken using identical laser settings and image acquisition parameters. One half of each panel also shows the anti-GCAP FITC fluorescence and nuclei counterstained with TO-PRO-3 iodide (*pseudo-blue*), superimposed on the DIC image of the same view field. Notice that the brightness of the anti-GCAP2 signal in the outer segment layer *versus* inner segment layer is slightly increased in the GCAP1^−/−^ retinas (marked with the dashed lines in *lower two panels*). **E.** Hematoxylin/eosin-stained GCAP1^−/−^ retinas at 6 months of age did not reveal evidence for retinal degeneration or other histological abnormalities when compared to the WT of the same age. Histological layers of the retina in **D** and **E** are marked as: *RPE*– retinal pigment epithelium, *OS*– photoreceptor outer segments, *IS*– inner segments, *ON*– outer nuclear layer, *OP*– outer plexiform layer, *IN*– inner nuclear layer, *IP* – inner plexiform layer, *GC* – ganglion cell layer. **F, G.** Representative electron micrographs of the WT and GCAP1^−/−^ ROS morphology: cross-sections (**F**; bar size –1 µm) and radial sections (**G**; bar size –0.2 µm); negative contrast by osmium.

The targeting construct for GUCA1A gene knockout (KO) was assembled in a pPNT vector harboring a *PGK:Neo:tts* cassette originating from Mulligan’s laboratory [23]. Long and short arms were amplified from mouse genomic clones (RP23-463A16 and RP23-184O9, CHORI BACPAC Resources, Berkeley, California) using a high-fidelity thermophylic Elongase polymerase (Invitrogen) and verified by DNA sequencing. The 5-kb long arm was amplified using 5′- AGGAAGGTACCGTCTGCAGT TACTTCTGGTTCCCATTGT-3′ and 5′- ACCGAACGCGTATTGTCTCAAACTCGA GGTCAGTAGTCA-3′ primers and ligated into the KpnI/MluI sites of the pPNT plasmid. The 1.2-kb short arm was amplified using 5′-AAAACGCGGCCGCATATAAGGACATTGG AAGAAGGGAGTGT-3′ and 5′-AAAAACCTGCAGGGAAAGAAAGCAAGAGGATC ATGAAATG-3′ primers and inserted into the SbfI/NotI sites of the plasmid harboring the long arm. The resultant construct was verified by restriction nuclease digestion and DNA sequencing was linearized by NdeI digestion, purified by Whatman Elutip minicolumn chromatography and concentrated by ethanol precipitation. The purified linearized construct was electroporated into B6/129SVE mouse hybrid embryonic stem cells (Ingenious Targeting Laboratory) and 288 clones were screened by PCR for homologous recombination of the short arm using 5′-TGGCTATGGATTCCAGAAGATTAAAACAGG-3′ (“f1” in **Fig. 1A**; 0.14 kb upstream from the short arm sequence in genomic DNA) and 5′-AGTGAGACGTGCTACTTCCATTTGTCA-3′ (“r1” in **Fig. 1A**, inside the *PGK:Neo:tts* cassette) primers. Seven positive clones identified in the primary screen were also verified for homologous recombination of the long arm by PCR using 5′-TGATATTGCTGAAGAGCTTGGCGGCGAAT-3′ primer (“f2”, inside PGK:Neo:tts cassette) and 5′-AAAAACGCGTGAACACAAACAGGCAGAAGTGAGGAGA-3′ (“r2”, 0.13 kb downstream from the long arm sequence in genomic DNA). Five knockout-positive clones were expanded and two of them were injected into mouse blastocysts (service was provided by Ingenious Targeting Laboratory). Clone 13G3 effectively passed the KO allele to the progeny and was used to establish a hemizygous, GCAP1^+/−^ line. After repetitive breeding to C57B6 WT mice (Taconic), GCAP1^+/−^ mice were crossed to produce the GCAP1^−/−^ genotype and the progeny were screened by PCR amplification from genomic tail DNA for the presence of the KO allele *versus* WT exon I using 5′-TCGGGAATCTTGCTTCATGGACATT-3′ and 5′- AGTGAGACGTGCTACTTCCATT TGTCA-3′ or 5′-CCTTGTGCAGGGGACATTA GAAAATAAG and 5′-CATCTGTTCCACATACTGGCTGGCT-3′ primers, respectively.

### Wild Type (WT) Mice and KO Hybrids

WT C57BL6 mice originated from Taconic. The double GCAPs1,2^−/−^ KO line, produced by simultaneous disruption of the neighboring *GUCA1A* and *GUCA1B* pair of genes ([14]), was a gift from Dr. Jeannie Chen (University of South California). The RetGC1^−/−^ (GC-E null) mice produced by the disruption of a mouse *GUCY2E* gene [24], was a gift from Dr. David Garbers (University of Texas), and RetGC2^−/−^ mice, produced by disruption of *GUCY2F* gene [25], were provided by Dr. Wolfgang Baehr (University of Utah). RetGC1^−/−^ and RetGC2^−/−^lines were crossed with GCAP1^−/−^ mice to produce RetGC1^−/−^ GCAP1^−/−^ and RetGC2^−/−^GCAP1^−/−^ genotypes, respectively.

*Antibodies* against full-size recombinant mouse GCAP1 and GCAP2 were raised in rabbits and purified on the corresponding immobilized GCAP affinity matrix [20]. Antibodies against human RetGC1 and RetGC2 were raised in rabbits immunized with 30 kDa recombinant fragments of the corresponding cyclases and purified on protein A Sepharose (GE Healthcare) as described [19]. Antibody against RGS9 was received from Dr. Vladlen Slepak (University of Miami), anti-arrestin 1 antibody was received from Dr. Vsevolod Gurevich (Vanderbilt University), and anti-GRK1 antibody 41072 was received from Dr. Jason Chen (Virginia Commonwealth University); anti-Gtα_1_ and anti-PDE6α antibodies were from AbCam, anti-β-actin – from GeneTex, and anti-rhodopsin – was from Chemicon/Millipore. Secondary goat anti-rabbit antibodies were conjugated with either horseradish peroxidase for immunoblotting (Pierce) or FITC (Cappel/MP Biomedical).

*Protein expression in the retina* was compared by SDS-gel electrophoresis and immunoblotting of retinal samples as described in [19], [22]. Blots were developed using a Pierce Femto Supersignal luminescent substrate kit (Thermo Scientific), and the chemiluminescence signals were acquired using a FotoDyne Luminous FX imaging system.

*GC activity* was assayed using [α-^32^P]GTP as a substrate [26]–[27] with modifications described in Peshenko et al. [19]. The resultant [^32^P]cGMP was analyzed using polyethyleneimine cellulose TLC, as described previously [28].

*Ca* ^2+^ */EGTA buffers* containing calibrated concentrations of free Ca^2+^ and Mg^2+^ were prepared using the method of Tsien and Pozzan [29] and verified by fluorescent Ca^2+^ indicator dyes as previously described [6].

### Electroretinography (ERG*)*

Scotopic ERG a-wave recovery was compared in different genotypes using the paired-flash approach [30] with minor modifications described in [22]. Mice were dark-adapted under a vented hood overnight and anesthetized by intraperitoneal injection of 20 µg/g Ketamine, 8 µg/g Xylazine, and 800 µg/g urethane. The pupils were fully dilated with 1% Tropicamide and Phenylephrine solutions applied topically 15 min prior to the recordings. During the recordings, mice were maintained on a heated plate. A 510 nm “test” flash injected into an integrating sphere delivered 5×10^3^ photons µm^−2^ at the cornea as a Ganzfeld, followed by an unfiltered white saturating “probe” flash delivering 5×10^5^ photons µm^−2^. The amplitude of the a-wave evoked by the probe flash was normalized for each inter-stimulus time interval by dividing by the amplitude of the response to the probe flash given prior to the conditioning test flash.

### Histology and Electron Microscopy

Following lethal injection of Ketamine/Xylazine mice were perfused through the heart, first with phosphate-buffered saline and then with freshly prepared 2.5% paraformaldehyde/2.5% glutaraldehyde mixture in phosphate-buffered saline. Enucleated eyes were then post-fixed in 2.5% glutaraldehyde/2.5% paraformaldehyde in sodium cacodylate buffer, pH 7.4 (Electron Microscopy Sciences), on ice for 4 hours, washed with 10 mM Na-phosphate/130 mM NaCl, pH 7.4, overnight, and processed for paraffin embedding (AML Laboratories). Sections, ∼3 µm in thickness, were stained with hematoxylin and eosine (AML Laboratories) and imaged using an Olympus BX51/Magnafire system. For electron microscopy, enucleated eyes were immersed in 2.5% glutaraldehyde/2% formaldehyde fixative and 0.08 M CaCl_2_ in 0.1 M cacodylate buffer for ∼24 hr at 4°C, washed with buffer and stored at 4°C. Eyes were post-fixed with 2% OsO_4_ for 90 min, dehydrated with ethanol, transitioned to propylene oxide and embedded in Epon resin. Sections from central retina were imaged on a Philips CM-10 transmission electron microscope and analyzed using ImageJ 1.42q (NIH) and PixelStick 1.1 (Plum Amazing, Princeville, HI). Measurements of the repeat distance for disks were made from arrays of 29 to 72 consecutive disks in rods whose disks were well organized. Rod diameter was determined from cross sections of rods with disks bearing an incisure.

### Immunofluorescence

Eyes from 4% paraformaldehyde-perfused animals were fixed on ice for 6 hours with 4% paraformaldehyde in phosphate-buffered saline, washed with 10 mM Na-phosphate/130 mM NaCl, pH 7.4, overnight, impregnated with 30% sucrose solution for 48 hours at 4°C, and then frozen in OCT media (Electron Microscopy Sciences). Cryosections were taken using a Hacker-Bright OTF600 cryomicrotome, probed with antibodies as described in [20], and viewed using an Olympus IBX81 microscope/FV1000 Spectral confocal system. Images were captured using Olympus FluoView FV10-ASW software. Where indicated, nuclei were counterstained with TO-PRO-3 (Invitrogen) and the fluorescence was superimposed on a differential interference contrast (DIC) image.

### Single Rod Recordings

Retinas from mice that were dark-adapted overnight were stored in chilled, oxygenated Leibovitz’s L-15 medium. Finely chopped pieces of retina were perfused with an enriched Locke’s solution equilibrated with 95% O_2_/5% CO_2_ at 37°C in an experimental chamber. The perfusion solution containing (mM): 139 Na^+^, 3.6 K^+^, 2.4 Mg^2+^, 1.2 Ca^2+^, 123.3 Cl^−^, 20 HCO_3_^−^, 10 HEPES, 3 succinate, 0.5 L-glutamate, 0.02 EDTA and 10 glucose, was supplemented with 0.1 mg/ml BSA (Fraction V, Sigma), 1% (v/v) MEM amino acids (Invitrogen), and 1% (v/v) BMEM vitamins (Sigma). A rod outer segment was pulled into a glass pipette and responses to flashes (nominally 3 ms in duration) were recorded with a patch clamp amplifier (Axopatch 200B, Axon Instruments, Union City, CA). Light from a shuttered, xenon source passed through a 500 nm interference filter and neutral density filters. The pipette was filled with HEPES buffered Locke’s solution lacking amino acids and vitamins, in which bicarbonate was replaced with Cl^-^. Photoresponses were low pass filtered at 30 Hz (−3 dB, 8-pole Bessel, Frequency Devices, Haverhill, MA) and digitized at 400 Hz on a MacIntosh computer (Pulse, version 8.31, HEKA Elektronik, Germany). Responses were also recorded with a SCSI based data acquisition system (CDAT4, Cygnus Technology, Delaware Water Gap, PA) for later re-digitization. No corrections were made for the delay of ca. 17 ms introduced by low pass filtering. Recorded data were digitally filtered by convolution with a Gaussian (Igor Pro version 5.04B, WaveMetrics, Lake Oswego, OR), which smoothed the waveform without introducing any delay.

## Results

### Disruption of the GUCA1A Gene Generates a GCAP1 Null Condition

Replacement of the first exon of the *GUCA1A* gene together with the 5′-upstream fragment of the putative promoter region with the *PGK:Neo:tts* cassette [23] containing a transcription termination site (*tts*) completely eliminated GCAP1 expression in mouse retinas (**Fig. 1A, B**). In immunoblotting samples standardized by rhodopsin concentration, GCAP1 signal was undetectable (**Fig. 1C**). The immunofluorescence in WT retinal cryosections probed with anti-GCAP1 polyclonal antibody was strong in rods and cones and was completely absent from GCAP1^−/−^ retinas (**Fig. 1D**). In contrast, anti-GCAP2 signal in GCAP1^−/−^ retinas not only remained clearly detectable, but was brighter than in the WT, particularly in the rod outer segments (**Fig. 1D**).

**Figure 2:**
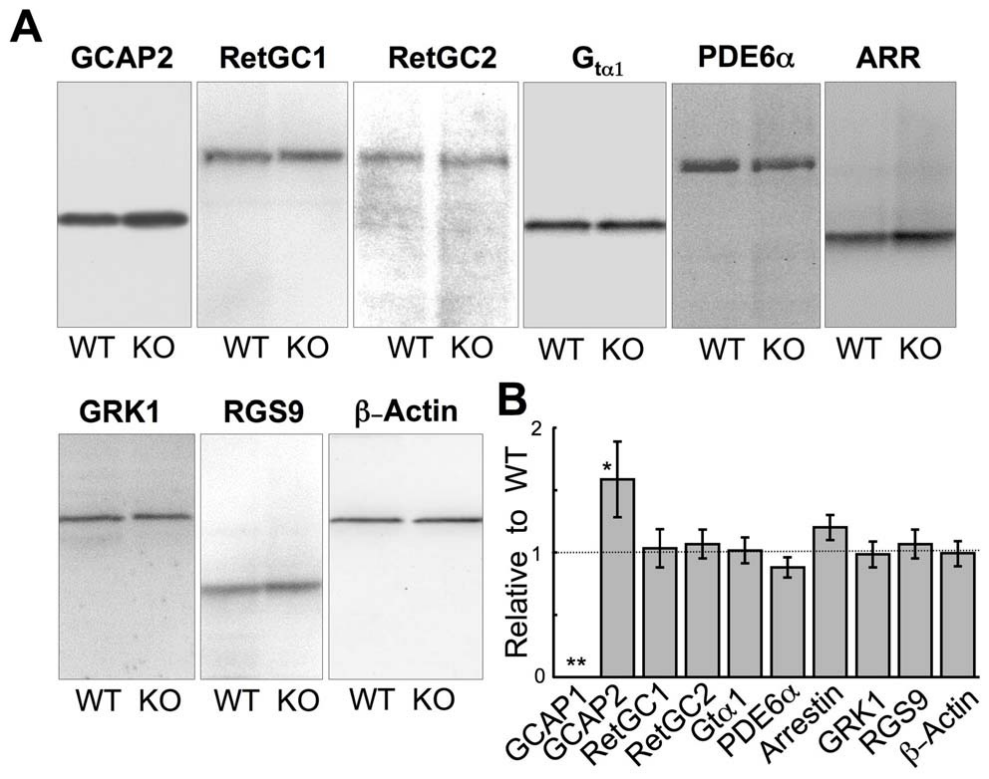
Photoreceptor protein expression in GCAP1^−/−^ retina. **A.** Immunoblots of SDS polyacrylamide gels containing samples from WT and GCAP1 KO retinas probed with antibodies raised against GCAP2, RetGC1, RetGC2, rod α-transducin (Gtα1), PDE6, arrestin 1 (ARR), GRK1, RGS9, and β-actin, as indicated. **B.** Average (± SD) integrated chemiluminescence signal intensity in the band for the corresponding antigen in GCAP1^−/−^ retina relative to the WT for GCAP1 (n = 5), GCAP2 (n = 7), RetGC1 (n = 3), RetGC2 (n = 3), rod α-transducin (n = 3), PDE6 (n = 3), arrestin (n = 3), GRK1 (n = 3), RGS9 (n = 3), and β-actin (n = 3). When compared by one-way ANOVA with Bonferroni’s *post hoc* test (alpha = 0.01), there were significant differences found in GCAP1 (**) and GCAP2 (*) contents (P<0.0001 and P<0.006, respectively), but not in other tested proteins.

The enhanced anti-GCAP2 immunofluorescence reflected an overall elevation of the GCAP2 expression levels in the retina as detected by immunoblotting - there was ∼60% more GCAP2 protein in KO compared to WT retinas (**Fig. 2**). Aside from the complete lack of GCAP1 (**Fig. 1C**), elevated GCAP2 expression was the only compensatory change among tested photoreceptor proteins that differed significantly in GCAP1^−/−^ retinas; there were no drastic changes in the expression levels of rod phototransduction cascade proteins: transducin, PDE6, arrestin 1, GRK1 and RGS9 **(** **Fig. 2** **)**. Most importantly, neither RetGC1 nor RetGC2 were strongly affected by knocking out GCAP1.

KO of GCAP1 did not cause a retinal degeneration or otherwise affect gross retinal morphology. The outer nuclear layer, consisting of the photoreceptor nuclei, was of normal thickness indicating that few if any rods had been lost over at least 6 months (**Fig. 1E**). At the electron microscopic level (**Fig. 2F, G**), ROS diameter was normal for GCAP1^−/−^ rods, but ROS length and disk repeat distance were slightly larger in GCAP1^−/−^ rods. Averaged results are given in **Table 1**.

**Table 1:** Rod outer segment morphology.

Parameter	WT	GCAP1^−/−^
ROS length, µm	24.2±0.3 (n = 120)	25.1±0.2 (n = 116, P = 0.011)
ROS diameter, µm	1.35±0.02 (n = 35)	1.30±0.01 (n = 74)
Disk repeat distance, Å	277±5 (n = 25)	337±2 (n = 36, P = 1e−18)

Measurements were made on rods from the central retina of 2 or 3 mice of each type, aged 2–3 months (representative sections are shown in **Fig. 2F, G**). Data are given as mean ± SEM, (number of rods measured, P-value from a t-test for values less than 0.05).

### Changes in Ca^2+^-sensitive Guanylyl Cyclase Activity in GCAP1^−/−^ Retinas

**Figure 3:**
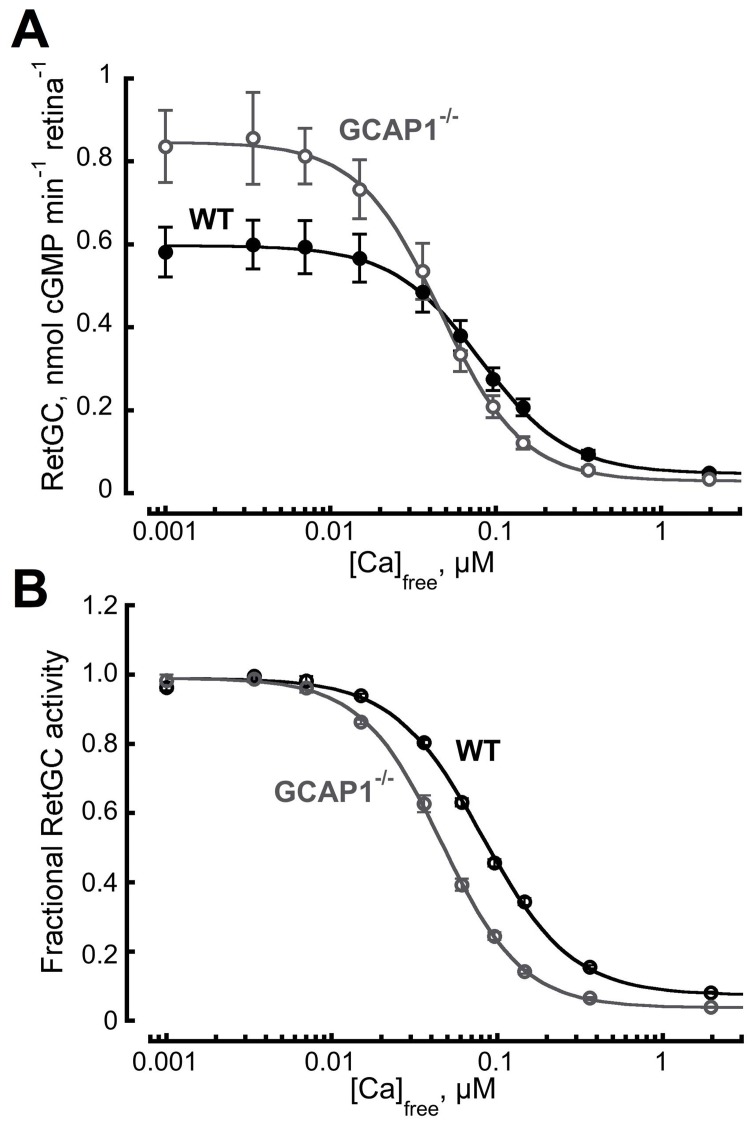
Altered RetGC activity in GCAP1^−/−^ mouse retinas. Total (**A)** and normalized (**B**) cGMP synthetic activity in WT (•, n = 5) and GCAP1^−/−^ (○, n = 4) retinas as a function of free Ca^2+^ concentration. Notice that sensitivity shifted to lower levels of Ca^2+^ in GCAP1^−/−^ retinas. In panel **B**, the activities in each series were normalized by the corresponding maximal RetGC activity measured in each genotype and averaged for each group. The data were fitted by the equation, *A = (A_max_ – A_min_)/(1+([Ca]/[Ca]_1/2_)^h^) + A_min_*; where *A_max_* and *A_min_* are the maximal and the minimal activity of guanylyl cyclase, respectively, *[Ca]_1/2_* is the concentration of Ca^2+^ producing 50% inhibition, and *h* is a Hill coefficient. RetGC activity was assayed as described in Materials and Methods. A_max_ values for the WT and GCAP1^−/−^ retinas were 0.6 and 0.8 nmol cGMP min^−1^ retina^−1^, [Ca]_1/2_ values were 81 and 46 nM, and *h* values were 1.8 and 1.6, respectively.

The maximal activity of RetGC at low [Ca^2+^] in GCAP1^−/−^ retinas was not diminished, but rather increased from 0.6±0.06 (mean ± SEM) nmol cGMP min^−1^ retina^−1^ in WT (n = 5) to 0.8±0.09 nmol cGMP min^−1^ retina^−1^ in the KO (n = 4) (**Fig. 3A**), apparently because of the compensatory increase in GCAP2 expression. Elimination of GCAP1 also changed the overall Ca^2+^-sensitivity of RetGC regulation in GCAP1^−/−^ retinas making it more sensitive to inhibition by Ca^2+^ (which is, less sensitive to activation by a decrease in free Ca^2+^). The [Ca]_1/2_ became significantly reduced (Student’s t-test P-value <0.0001), from 81±2 (mean ± SEM, n = 5) nM to 46±2 nM (n = 4), respectively (**Fig. 3B**).

### GCAP2 Provides Ca^2+^ Feedback to both RetGC1 and RetGC2 Isozymes in GCAP1^−/−^ Rods

**Figure 4:**
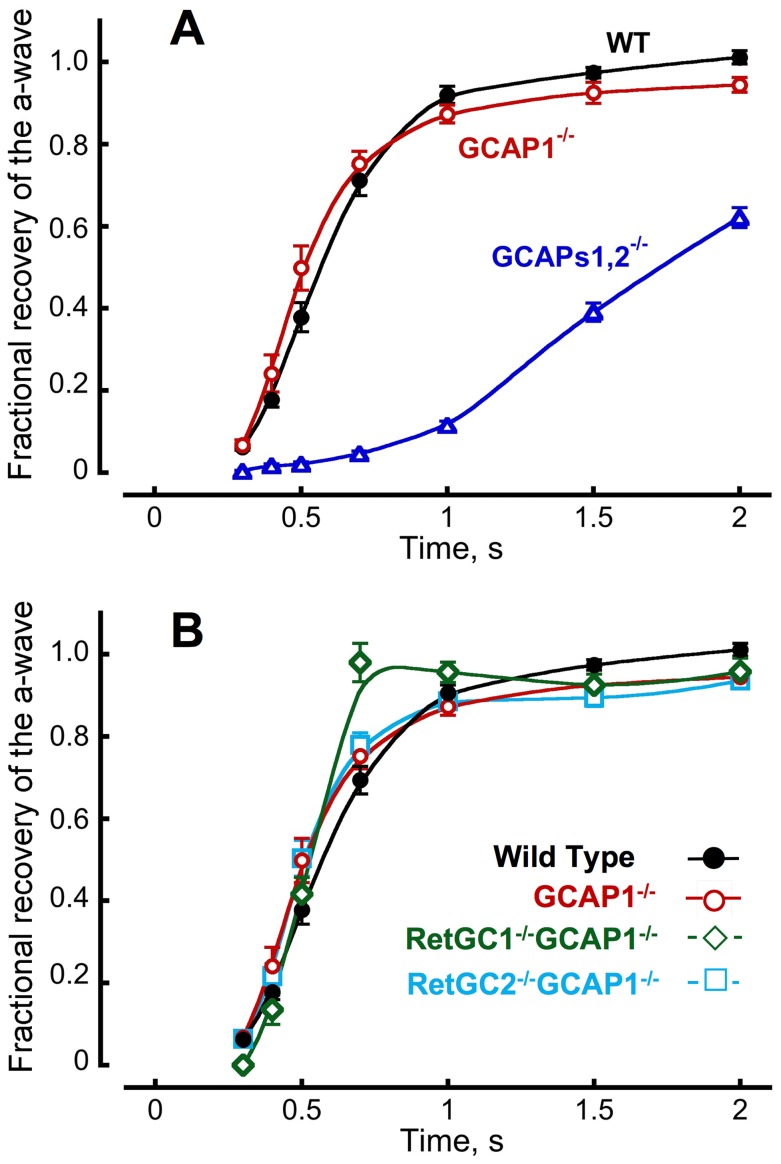
Recovery of bright flash response of rods, reconstructed from recordings of paired-flash ERGs. **A.** Fractional a-wave recovery from a strong flash, presented at time zero, in paired-flash ERGs from 16 WT (•) [22], 17 GCAPs1,2^−/−^ (▵), and 17 GCAP1^−/−^ (○) mice aged 1.5–3 months. **B.** The recovery remained fast in the absence of each RetGC isozyme; 16 WT (•), 17 GCAP1^−/−^ (○), 18 RetGC1^−/−^GCAP1^−/−^ (⋄), and 17 RetGC2^−/−^GCAP1^−/−^ (□) mice. The average saturating a-wave amplitudes in WT, GCAP1^−/−^, GCAPs1,2^−/−^, RetGC1^−/−^GCAP1^−/−^, and RetGC2^−/−^GCAP1^−/−^ were 532, 347, 365, 98, and 277 µV, respectively. The continuous curves were ‘smooth line’ fit by KaleidaGraph software. In many cases, only the initial phase of the ERG recovery could be fit by a single exponential. The time required for 50% amplitude recovery determined from the exponential portion of the fit in 16 mice for each genotype was (mean ± SEM): 0.55±0.02, 0.51±0.02, 0.50±0.02, 0.51±0.02, and 1.78±0.06 s in WT, GCAP1^−/−^, RetGC1^−/−^GCAP1^−/−^, RetGC2^−/−^GCAP1^−/−^, and GCAPs12^−/−^, respectively. In all-pairs comparison, the only significant difference for the entire group (P<0.0001, one way ANOVA with a Bonferroni post-hoc test, alpha = 0.01) was found between GCAPs1,2^−/−^ and all other genotypes. Contribution of a small fraction [45] of mouse cones to the scotopic a-wave amplitude was considered insignificant for this analysis.

The high cyclase activity in GCAP1^−/−^ retinas measured *in vitro* (**Fig. 3**) suggested that GCAP2 activated both RetGC1 and RetGC2 isozymes at low Ca^2+^concentrations typical of light-exposed rods [31]. For verification *in vivo*, we compared the rates of scotopic a-wave recovery in mice lacking GCAP1 and one of the two RetGC isozymes using a double-flash ERG paradigm [32]. The ERG is an extracellular field potential induced by the electrical activity of the retina as it responds to light. The corneal negative a-wave is generated by the photoreceptors. Since cones comprise only a minor fraction (∼3%) of photoreceptors and the cone ERG a-wave amplitude is negligible compared to the scotopic rod ERG a-wave in mouse, this experiment monitors almost exclusively rod activity. In our double-flash paradigm, the a-wave amplitude was measured in response to a saturating test flash. However, the true photocurrent responses of the rods were masked in the ERG by the activity of other retinal neurons. So a probe flash was then delivered. The probe flash was also saturating, but gave rise to a smaller a-wave if delivered at inter-flash intervals too short for the rod photocurrent response to the test flash to fully recover. By varying the delay between test flash and probe flash in separate trials, it was possible to reconstruct the time course of the rod photocurrent response to the test flash. Individual GCAPs1,2^−/−^ double KO rods recover much more slowly after bright flashes than WT rods [14]–[15], therefore not surprisingly, the GCAPs1,2^−/−^ ERG recovery was also prolonged (**Fig. 4A**). However, there was no such prolongation in GCAP1^−/−^ retinas. If anything, their rods recovered slightly faster, albeit showing a slow tail at the final stage of the recovery. The origin of the tail will be discussed later in this publication. There was no prolongation observed in RetGC1^−/−^GCAP1^−/−^ or RetGC2^−/−^GCAP1^−/−^ double KO mice, either (**Fig. 4B**). The responses recovered to 50% at 0.55, 0.51, 0.50, and 0.51 s after the flash in WT, GCAP1^−/−^, RetGC1^−/−^GCAP1^−/−^, and RetGC2^−/−^GCAP1^−/−^, respectively – more than 3 times faster than in GCAPs1,2^−/−^ mice (1.78 s, P<0.0001). Hence, the remaining GCAP2 efficiently activates both cyclase isozymes via negative Ca^2+^ feedback in living GCAP1^−/−^ rods.

### Elimination of GCAP1 Alters Rod Responses to Flashes

**Figure 5:**
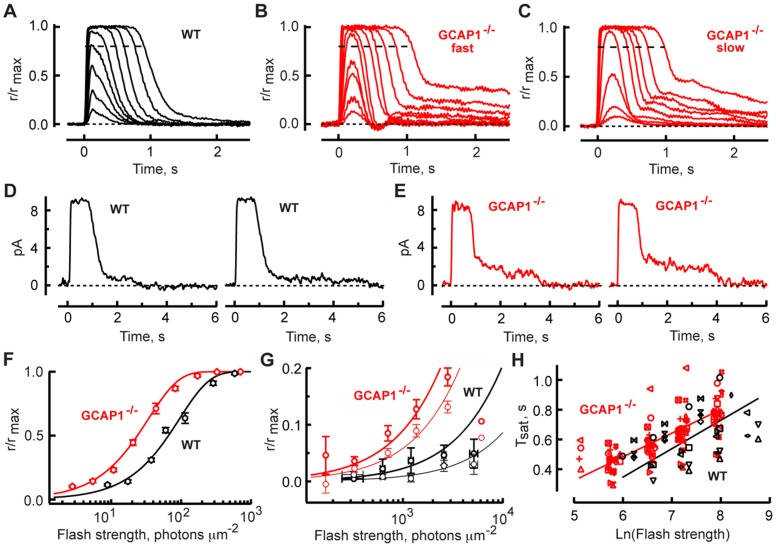
Changes in flash responses after deletion of GCAP1. Averaged flash responses of a WT rod (**A**) peaked sooner and had a reduced tail component in the recovery compared to two GCAP1^−/−^rods designated arbitrarily as having “fast” (**B**) or “slow” (**C**) response kinetics (marked accordingly as “fast” and “slow” in the panels). Maximal response amplitudes were 11, 10 and 14 pA, respectively. The integration times of dim flash responses, whose amplitudes were less than 20% of the maximal response, were 250 ms for the WT rod and 236 and 483 ms for the two GCAP1^−/−^ rods. The flash was presented at time zero. Flash strengths were: 14, 31, 58, 121, 227, 505, 945, 1973 and 3691 photons µm^−2^ for the WT rod; 6, 11, 23, 44, 91, 171, 380, 713, 1482, 2773 and 6091 photons µm^−2^ for the GCAP1^−/−^ rod in **B** and 3, 5, 20, 80, 311, 692, 1300, 2691 and 5045 photons µm^−2^ for the GCAP1^−/−^ rod in **C.** Records were digitally filtered at 12 Hz. **D.** Stepped recovery of the bright flash response in two trials for the WT rod in **A** due to aberrant photon responses. Flash strength was 3691 photons µm^−2^. The number of steps and the temporal depth of their tread varied randomly from trial to trial. **E.** Tendency for steps to be larger in GCAP1^−/−^ rods. Responses were recorded from a GCAP1^−/−^ rod different from those in **B** and **C.** Flash strength was 2773 photons µm^−2^. Records were digitally filtered at 8 Hz. **F.** Average stimulus-response relations for 28 WT (*black*) and 36 GCAP1^−/−^ (*red*) rods. Each circle averages the normalized responses of several rods that were grouped by similar flash strength, and error bars show SEM. Continuous lines show the saturating exponential function *r/r_max_ = 1− exp(-ki)*, where *i* is flash strength, *k* is equal to ln(2)/i_0.5_, and *i_0.5_* is the flash strength that produces a half-saturating response, with i_0.5_ values of 66 and 23 photons µm^−2^ for WT and GCAP1^−/−^, respectively. These i_0.5_ values were derived from the mean *k* from fits to individual WT and GCAP1^−/−^ rods. **G.** Stimulus-response relations for the tail of saturated responses from 16 WT (*black*) and 35 GCAP1^−/−^ (*red*) rods, measured at 1.5 (*thick symbols*) and 2 s (*thin symbols*) after the flash. Each symbol represents the average, normalized response amplitude of 12 to 15 WT rods or 24 to 30 KO rods (except at the lowest and highest flash strengths, for which there were only 1–6 rods), where groups were made according to flash strength. Error bars for flash strength are shown although variation was negligible on a log scale. Continuous lines show saturating exponential functions with averaged values for *k* (see above) derived from fits to individual rods. **H.** Pepperberg plot [34] for 11 WT (*black*) and 28 GCAP1^−/−^ (*red*) rods. The saturation time of a bright flash response was measured from mid-flash to the point at which the saturation response declined to 0.8 r_max_, i.e., 20% recovery, as demarcated by the dotted lines in **A–C**. Results from each rod were plotted with a different symbol. The continuous lines have slopes equal to τ_c_ of 191 ms for WT and 159 ms for GCAP1^−/−^, that were the mean values of linear regressions from individual rods in each group (**Table 2**).

In electrical recordings of single rods, elimination of GCAP1 increased sensitivity to flashes by more than two-fold and saturated the rod at lower intensities (**Fig. 5A–C, F**). With bright flashes, there was a long-lived tail in the response. Tails were also present in saturating responses of WT rods (e.g. the response to the brightest flash in **Fig. 5A**). In individual trials, tails recovered in randomly spaced, “stepped” transitions back to the baseline (**Fig. 5D**) and were attributed to rare, enlarged photon responses of aberrantly long duration [33]. Tails in KO rods were similar except that they were more pronounced and even appeared in responses to some subsaturating flashes (**Fig. 5B, C, E**). Stimulus-aberrant response relations for the tails of flash responses measured 1.5 and 2 s postflash were shifted to four- to seven-fold lower flash strengths in GCAP1^−/−^ (**Fig. 5G**). This shift is even greater than that observed in the stimulus-response relations for the peaks of the flash responses (**Fig. 5F**), suggesting that aberrant SPRs were larger in GCAP1^−/−^ rods although we cannot at this time rule out a greater frequency, a longer duration or some combination of effects.

**Table 2:** Rod photoresponse parameters in WT and GCAP1^−/−^ mice.

Parameter	WT	GCAP1^−/−^
i_0.5_, photons µm^−2^	79±6 (n = 28)	26±1 (n = 36, P = 3e−13)
Single-photon-response amplitude, pA	0.45±0.05 (n = 18)	1.02±0.10 (n = 36, P = 3e−4)
Time to peak, ms	144±5 (n = 22)	244±10 (n = 37, P = 4e−10)
Integration time, ms	352±44 (n = 22)	309±19 (n = 37)
Recovery time constant, τ_r_, ms	240±20 (n = 22)	119±13 (n = 37, P = 2e−6)
Saturation time constant, τ_c_, ms	191±10 (n = 11)	159±7 (n = 28, P = 2e−2)
R_max_, pA	8.9±0.4 (n = 34)	9.3±0.2 (n = 54)
Fractional amplitude	0.046±0.005 (n = 18)	0.104±0.008 (n = 36, P = 1e−5)

**Figure 6:**
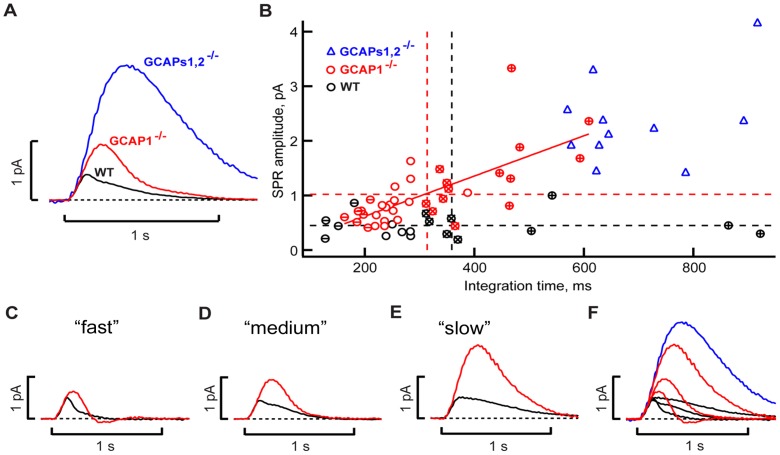
Heterogeneity in WT and GCAP1^−/−^ rods. **A.** The dim flash response, whose amplitude was less than 20% of the maximal response, was scaled to the amplitude of the SPR for each rod and averaged for 18 WT (*solid black trace*), 36 GCAP1^−/−^ (*red trace*), and 11 GCAPs1,2^−/−^ (*blue trace*) rods. Traces were digitally filtered at 12 Hz. Although the SPR amplitude and time-to-peak of GCAP1^−/−^ rods were twice those of WT, the averaged response of GCAP1^−/−^ could not reflect the wide range of characteristics of the group. **B.** Increase in the SPR amplitude with integration time for GCAP1^−/−^ rods (Ο, *red*) but not for WT rods (○, *black*) or for GCAPs1,2^−/−^ rods (Δ, *blue*). Dotted horizontal and vertical lines demarcate the mean SPR amplitudes and integration times, respectively for WT (*black*) and GCAP1^−/−^ rods (*red*). *Solid red line* was linear fit for GCAP1^−/−^ rods; the Pearson product-moment correlation coefficient was 0.71. **C–F**, SPRs for selected groups of WT (*black*) and GCAP1^−/−^ (*red*) rods that were designated arbitrarily as having fast (**C**), medium (**D**) or slow (**E**) integration times. The rods with fast, medium and slow integration times have symbols marked with “**–**”, “**×**” and “**+**” in **B**, respectively. Responses from all groups were gathered in **F**, along with that of GCAPs1,2^−/−^ (from **A**). For WT rods, times to peak were 138, 135 and 163 ms for groups with fast, medium and slow integration time, respectively, but the SPR amplitudes remained similar: 0.5, 0.4 and 0.5 pA. For GCAP1^−/−^ rods, the mean SPR times-to-peak were 195, 225 and 333 ms and the SPR amplitudes were 0.6, 0.9 and 1.8 pA, respectively. The SPR in GCAPs1,2^−/−^ rods had a time-to-peak of 380 ms and an amplitude of 2.3 pA.

Parameters for both WT and GCAP1^−/−^ mice average all rods of each type and include “fast” and “slow” rods (see **Figures 5** and **6** and the *Discussion* section). Results are given as mean ± SEM (number of cells recorded, P-value from a Student’s t-test for values less than 0.05). The i_0.5_ is the flash strength at 500 nm eliciting a half-maximal response, and it varies inversely with sensitivity. SPR amplitude was estimated by dividing the ensemble variance by the mean dim flash response amplitude. Kinetics of the single-photon response were determined from dim flash responses whose amplitude was less than 20% of the maximum. Time to peak was measured from mid-flash to the response peak. Integration time was calculated as the integral of the response divided by response amplitude. Recovery time constant, τ_r_, refers to a fit of the final falling phase of the dim flash response with a single exponential. Saturation time constant, τ_c_, is the slope of the relation between saturation time and the natural logarithm of the flash strength, by linear regression. R_max_ is the maximum circulating current recorded from a rod, and fractional amplitude is taken as a ratio of the single-photon-response amplitude to the maximum circulating current from that rod.

In WT rods, response saturation time increases linearly with the natural logarithm of the flash strength and the slope of the relation, τ_c_, estimates the shutoff rate of the slowest cascade component [34], namely that of the transducin/PDE complex [35]. Although saturation time and the natural logarithm of the flash strength were still linearly related in GCAP1−/− rods there was an upward shift as would be expected from their higher sensitivity (**Fig.** **5H**). In addition, the mean τ_c_ was slightly faster than for WT rods. One likely explanation is that τ_c_ was distorted by altered RetGC activity. GCAP1^−/−^ rods may have taken longer to reach maximal RetGC activity because GCAP2 has a higher affinity for Ca^2+^ and therefore requires Ca^2+^ to drop lower before it fully activates the cyclase. Thus at lower flash strengths, for which response saturation times were relatively short, RetGC may not yet have attained maximal activity and emergence from saturation was delayed. But with longer saturation times, GCAP2 fully activated the cyclase to a level that was more powerful than normal accelerating recovery from saturation. The net effect was a decrease in τ_c_.

In addition, there were profound changes in the shape of the GCAP1^−/−^ responses to dimmer flashes (**Figs. 5A–C** **and** **6** **,** **Table 2**). On average the single-photon response for GCAP1^−/−^ rods showed increased amplitude, delayed time to peak, and accelerated recovery (**Table 2**). Although an average SPR is shown in **Fig. 6A**, it needs to be emphasized that such a waveform was never observed in any particular rod because the shapes of SPRs in individual GCAP1^−/−^ rods were highly variable. There was a continuum of response waveforms falling between two extremes. At one extreme, flash responses from “fast” rods took longer to reach a peak and then recovered very quickly, overshooting the baseline (**Figs. 5B** **,** **6C**). At the other extreme, “slow” rods tended to be more sensitive, flash responses peaked even later and the recovery was somewhat slower than in fast cells, but still faster than in WT rods (**Figs. 5C** **,** **6E** **)**. The overshoot of the baseline was missing in these slow cells. Unlike WT rods, the amplitude of the GCAP1^−/−^ response rose in proportion to the integration time (**Fig. 6B**). Nevertheless, when compared to WT rods with a similar integration time, all GCAP1^−/−^ rods showed higher amplitudes and faster recovery kinetics (**Fig. 6B, C–F**). In contrast, photon responses of GCAPs1,2^−/−^ double KO rods recovered quite slowly and clustered around long integration times (**Fig. 6A, B**). Fast and slow rods were encountered within the retinas of eight of the nine GCAP1^−/−^ mice studied, for which three to nine rods were recorded per retina. In the remaining mouse, one fast rod and four medium rods were recorded. These changes were generally consistent with the altered Ca^2+^ sensitivity of the cyclase regulation, but the variability suggested that GCAP2 content varied considerably between GCAP1^−/−^ outer segments. More subtle differences in GCAP2 content in WT rods may have contributed to the variability in their integration times (**Fig. 6B–F**).

## Discussion

**Figure 7:**
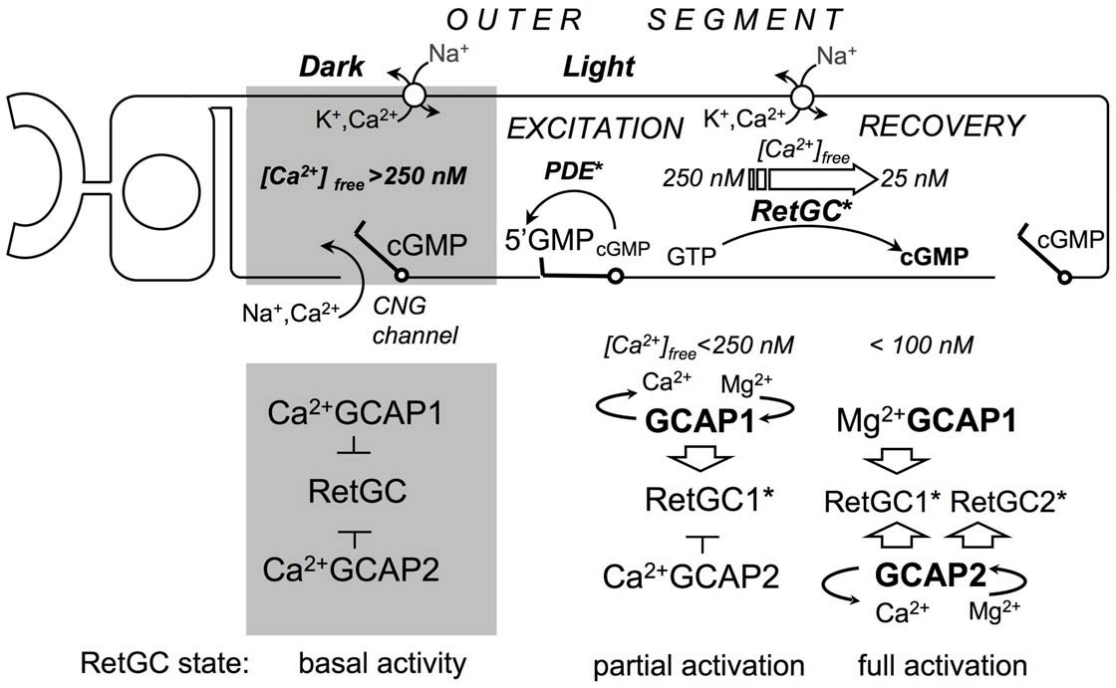
Two-step relay mechanism [14] [20]–[21], for cGMP synthesis regulation in rods by GCAP1 and GCAP2. Free Ca^2+^ in rod outer segment is maintained by an efflux through a constitutively active Na^+^/K^+^, Ca^2+^ exchanger and an influx through the cyclic nucleotide gated (CNG) channels. In the dark, when the CNG channels are open, the intracellular free Ca^2+^ concentrations are relatively high, so both GCAP1 and GCAP2 bind Ca^2+^ and inhibit cGMP synthesis. Once the PDE6 cascade becomes activated by a bright flash, cGMP decays, CNG channels close, Ca^2+^ influx through the CNG channels stops and the concentration of free Ca^2+^ starts to fall. GCAP1 responds first by converting to a Mg^2+^-bound [46] activator state and accelerates cGMP re-synthesis, thus limiting the number of the closed CNG channels and suppressing the amplitude of a dim flash response. GCAP2 has higher affinity for Ca^2+^ and therefore remains Ca^2+^ bound longer than GCAP1, but as free Ca^2+^ continues to drop at the peak of the response, GCAP2 also converts to the activator form and provides additional stimulation of the cyclase in mid-phase of the recovery thus accelerating its kinetics. Based on the *in vivo* target enzyme specificity of GCAP1 for RetGC1 [22] and the ambivalent target enzyme specificity of GCAP2 (**Fig. 4**), RetGC1 becomes the ‘first-response’ cyclase isozyme, activated by GCAP1 early in response, while both RetGC1 and RetGC2 would then be additionally activated by GCAP2 in mid-phase of the response to speed up the recovery.

Previous studies using GCAPs1,2^−/−^ double KO mice, in which a portion of the chromosome coding for both GCAP isoforms was deleted, established that Ca^2+^ feedback to the cyclase is essential for the normal shape of the rod photoresponse [14], [15]. Transgenic overexpression of either GCAP2 or GCAP1 in the GCAPs1,2^−/−^ rods [14], [36] accelerates slow responses of GCAPs1,2^−/−^. However, the two GCAP isoforms are not merely redundant Ca^2+^ sensors for RetGC regulation. GCAPs, due to their different Ca^2+^ sensitivities [17]–[19] activate RetGC during the photoresponse sequentially, in a relay fashion, described in **Figure 7**. GCAP1, which requires higher concentrations of Ca^2+^ to suppress its ability to activate the cyclase, starts RetGC activation as Ca^2+^ concentrations begin to decline soon after photoexcitation, and therefore limits the amplitude of a single-photon response, while GCAP2 does not contribute to RetGC acceleration until Ca^2+^ concentrations fall to their minimal levels after the response peak so it serves to quicken the SPR recovery. Even though the two GCAPs have different specific effects on the SPR, both result in a shift of the rod’s operating range to higher intensities.

The relay model of sequential acceleration of RetGC activity accounts for the results of *in vitro* studies of Ca^2+^ sensitivity of GCAP1 and GCAP2 [17]–[18] as well as the biochemical and physiological properties of RetGC regulation in GCAP2^−/−^ rods [20], where the [Ca]_1/2_ of RetGC inhibition rose two-fold as a result of elimination of GCAP2, and in GCAP1^−/−^ rods in our present study, where Ca^2+^ sensitivity shifted two-fold in the opposite direction, making RetGC more sensitive to inhibition by Ca^2+^ than normal (**Fig. 3**). The observed change in Ca^2+^ sensitivity of the retinal cGMP synthesis in both KO models is consistent with the difference in Ca^2+^ sensitivity of mouse GCAP1 and GCAP2 observed *in vitro* [19]. Single rod responses drastically changed in GCAP1^−/−^ in a manner generally consistent with loss of the ‘first-response’ Ca^2+^ sensor (**Figs. 5** **,** **6** **,** **Table 2**). Photon responses rose to a larger amplitude and peaked ∼110 ms later than normal, evidently because cyclase activity failed to accelerate in response to the initial decline in free cytoplasmic Ca^2+^ concentration that occurred soon after CNG channel closure. While these findings, together with the previous observations [14], [20], strongly support the relay model of RetGC regulation *in vivo* (**Fig. 7**), they also revealed several unexpected phenomena.

Deletion of GCAP1 might have been expected to slow flash response recovery, yet recovery kinetics in GCAP1^−/−^ rods remained fast, typically faster than in WT rods (**Table 2**, **Figs. 4** **,** **5** **,** **6**). Apparently, in the absence of GCAP1, the more Ca^2+^-sensitive GCAP2 isoform took over the entire regulation of RetGC in photoreceptors, because GCAP1^−/−^ retinas expressed greater GCAP2 protein levels (**Fig. 2**) compensating for the lack of GCAP1. Anti-GCAP2 immunofluorescence signal was visibly brighter indicating a higher concentration of GCAP2 in GCAP1^−/−^ rod outer segments (**Fig. 1D**), and maximal RetGC activity in GCAP1^−/−^ retinas increased (**Fig. 3**). The up-regulation of GCAP2 in GCAP1^−/−^ retinas suggests that either transcriptional or translational regulation elevated GCAP2 synthesis in the absence of GCAP1, a phenomenon that deserves special study. Interestingly, there was no significant up-regulation of GCAP1 observed in GCAP2^−/−^ retinas and flash recovery did slow down [20].

For maximal activity to increase, GCAP2 must have taken over the regulation of both RetGC1 and RetGC2 isozymes. Both GCAP1 and GCAP2 are capable of activating mouse RetGC1 and RetGC2 isozymes in native mouse ROS membranes *in vitro* [19], although GCAP1 *in vivo* preferentially activates RetGC1 [21]. GCAP/RetGC complexes cannot be analyzed biochemically, because detergents required for extraction of RetGC from the membrane destroy GCAP/RetGC interactions [37], yet GCAP1^−/−^ retinal biochemistry (**Fig. 3**) and physiology (**Fig. 4**) both argue that GCAP2 not only activates the two RetGC isozymes *in vitro* but also maintains complexes with both of them in living photoreceptors. GCAP1, on the other hand, accelerates RetGC2 *in vitro* but fails to do so *in vivo* [22] likely because of presently unidentified cellular sorting mechanisms rather than its intrinsic biochemical properties [19]. Since RetGC1 is the preferential target for GCAP1 *in vivo* [22], RetGC1 is then the ‘first response’ cyclase isozyme required for early suppression of the rod response amplitude by partial acceleration of cGMP re-synthesis (**Fig. 7**), while both RetGC1 and RetGC2 can become fully activated in mid-phase of the recovery. Additional study into the mechanisms of selectivity underlying GCAP/RetGC interaction *in vivo* will be required to establish their role in shaping the photoresponse.

Even with the compensation by the increased levels of GCAP2 in rods (**Figs. 1** **,** **2**), the RetGC activity measured in GCAP1^−/−^ retinas (**Fig. 3**) appeared to be higher than expected. Although GCAP2 is capable of activating both RetGC isozymes *in vitro* [19] and in the living rods (**Fig. 4**), recombinant mouse GCAP2 stimulates native RetGC isozymes to a lower maximal activity compared to the recombinant mouse GCAP1 *in vitro* [19]. Since neither RetGC1 nor RetGC2 expression underwent a dramatic change in the GCAP1^−/−^ retinas (**Fig. 2**), the higher levels of the cyclase activity could indicate that either the native GCAP2 present in outer segments stimulates RetGC more efficiently than the recombinant GCAP2 or that normally, GCAPs do not fully saturate RetGCs *in vivo*.

Photon responses were highly variable in amplitude and duration between individual GCAP1^−/−^ rods within the same retina (**Figs. 5A–C** **,** **6**). This likely reflects variable levels of GCAP2 expression between different rods in GCAP1^−/−^ mice. Interestingly, transgenic overexpression of GCAP2 in GCAPs1,2^−/−^ rods driven by a rhodopsin promoter accelerates recovery after a strong flash and produces similar rod to rod variability in the dim flash response [14]. While variations in transgenic rhodopsin promoter activity [38]–[39] may have been responsible, our experiments argue that GCAP2 could be constitutively expressed at different levels even between rods of a WT mouse and that those differences may simply be accentuated in GCAP1^−/−^ retinas, either by a specific mechanism regulating the level of GCAPs expression or by our having inserted a construct containing PGK promoter in the vicinity of the chromosome region coding for GCAP2.

The overshoot in response recovery, frequent in WT rods containing BAPTA, e.g. [15], also appeared in many GCAP1^−/−^ rods (**Fig. 5**). This suggests that GC activity did not attenuate quickly after the CNG channels reopened. Considering that GCAP2 has higher sensitivity to Ca^2+^ than GCAP1 [19], that cGMP and Ca^2+^ are believed to equilibrate rapidly within mammalian outer segments in the transverse direction (cf. [40]–[41]) and that Ca^2+^ affects cGMP synthesis in homogenized ROS without a biochemically detectable lag phase [42], this effect is somewhat puzzling. However, a standard biochemical assay cannot provide sufficient resolution on a millisecond scale. At the same time, the spatial and structural organization of GCAP/RetGC complexes within the outer segment remains poorly understood. Alternatively, we cannot completely exclude that the exchange of Mg^2+^ for Ca^2+^ in EF-hands of GCAP2 bound to the cyclase *in vivo* occurs after a short delay. If GCAP2 levels approach 10 µM in the GCAP1^−/−^ ROS as it was estimated for wild type [19], it could also buffer internal Ca^2+^ [14], causing a small temporal lag between the influx of Ca^2+^ and cessation of RetGC stimulation by GCAP2 in intact rods. According to the model in Figure 7, we would expect that GCAP2 is the first sensor to be turned off at the end of the recovery, unless any delay in Ca^2+^ effect on GCAP2 at the end of the recovery makes both GCAPs become turned off nearly at the same time.

In mammalian rods, approximately one photoisomerization of rhodopsin out of several hundred gives rise to an aberrant response that rises to an amplitude that is 1.5−2× larger than normal and persists for an unpredictable period of time, lasting on average for about 3–4 seconds [33], [43]–[44]. Aberrant responses are caused by improper phosphorylation and shutoff of photoexcited rhodopsin [43]–[44]. In GCAP1^−/−^ rods, the aberrant responses appeared to be enlarged, underscoring the importance of GCAP1 in suppressing their size. It is not yet clear whether they also had a longer duration or occurred more frequently. The consequence of knocking out the “first response” Ca^2+^ sensor, GCAP1, even in the face of overexpression of GCAP2, was that the recovery after exposure to bright light was inordinately long.
